# Combat as an Interpersonal Synergy: An Ecological Dynamics Approach to Combat Sports

**DOI:** 10.1007/s40279-019-01173-y

**Published:** 2019-09-09

**Authors:** Kai Krabben, Dominic Orth, John van der Kamp

**Affiliations:** 1 Department of Human Movement Sciences, Faculty of Behaviour and Movement Sciences, Vrije Universiteit Amsterdam, Amsterdam Movement Sciences, Van der Boechorststraat 9, 1081 BT Amsterdam, The Netherlands; 2 Department of Health and Medical Sciences, Swinburne University of Technology, Melbourne, Australia

## Abstract

In combat sports, athletes continuously co-adapt their behavior to that of the opponent. We consider this interactive aspect of combat to be at the heart of skilled performance, yet combat sports research often neglects or limits interaction between combatants. To promote a more interactive approach, the aim of this paper is to understand combat sports from the combined perspective of ecological psychology and dynamic systems. Accordingly, combat athletes are driven by perception of affordances to attack and defend. Two combatants in a fight self-organize into one *interpersonal synergy*, where the perceptions and actions of both athletes are coupled. To be successful in combat, performers need to manipulate and take advantage of the (in)stability of the system. Skilled performance in combat sports therefore requires *brinkmanship*: combatants need to be aware of their action boundaries and purposefully act in meta-stable regions on the limits of their capabilities. We review the experimental literature to provide initial support for a synergetic approach to combat sports. Expert combatants seem able to accurately perceive action boundaries for themselves and their opponent. Local-level behavior of individual combatants has been found to lead to spatiotemporal synchronization at the global level of a fight. Yet, a formal understanding of combat as a dynamic system starting with the identification of order and control parameters is still lacking. We conclude that the ecological dynamics perspective offers a promising approach to further our understanding of skilled performance in combat sports, as well as to assist coaches and athletes to promote optimal training and learning.

## Key Points

**Table:** 

A review of the literature on skilled behavior in combat sports shows initial support for conceptualization of combat dyads as a single dynamical system or *interpersonal synergy*.
This position implies that skilled behavior should not be sought solely within the individual athlete, but rather that the emergence of skilled performance and learning is distributed across the athlete–opponent interaction.
Combat athletes and coaches should seek to develop ‘brinkmanship’ to purposefully and accurately perceive and act near their action boundaries.

## Introduction

In combat or fighting sports, two athletes engage in a regulated form of one-on-one combat in which they attempt to strike, throw, and/or submit the opponent combatant using a range of different offensive and defensive actions. In doing so, combat athletes need to continuously co-adapt their behavior to that of the opponent in a constant game of anticipation, action, and re-action [1–3]. This highly dynamic interaction provides an intriguing but very challenging area for the study of perception, action, and cognition [1, 4]. Although research on perceptual–motor expertise in combat sports has advanced over the last decades, most empirical work has largely neglected or limited the complex interpersonal aspects of combat. Researchers have mostly focused on a single combatant within an artificially controlled environment [5, 6] or on two combatants with set roles of attack and defense [1, 7]. These approaches, although potentially insightful into some aspects of skilled performance and learning, fail to fully capture the complex and inherent interactive richness of behaviors that characterize one-on-one combat situations.

**Fig. 1:**
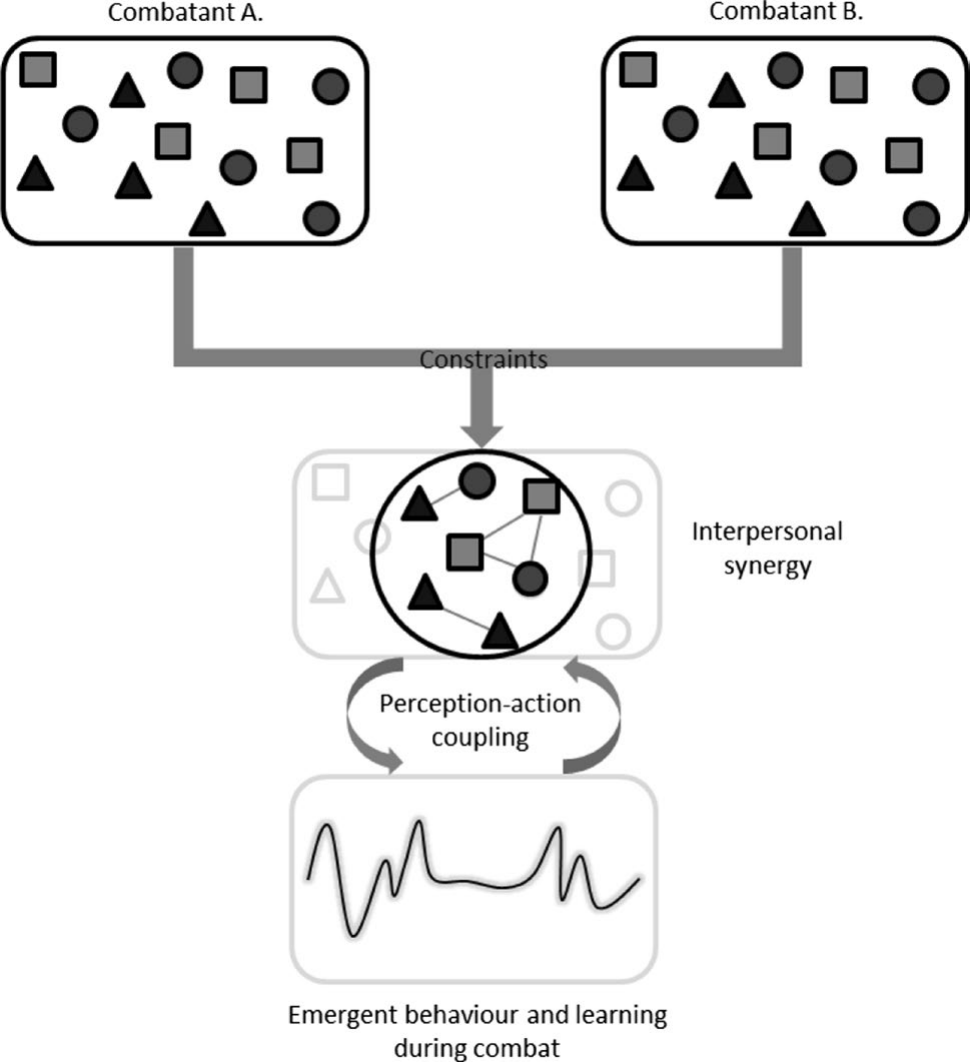
Conceptualization of combat as an interpersonal synergy adapted from Riley et al. [13]. Behavior and learning emerge during combat from reciprocal couplings between the two individual combatants’ perception and action

More recently, a few researchers have started to study combat sports in more interactive and engaged contexts [4, 8, 9]. In this paper, we aim to further promote this outlook by understanding combat interaction from the combined perspectives of ecological psychology and dynamic systems, which we refer to as the ecological dynamics approach [10–12]. Our main proposal is that by agreeing to compete in a one-on-one combat situation, the two athletes form what has been coined an interpersonal synergy [13, 14]. In a one-on-one combat situation, the behavior of combatant A is directly interdependent on and constrained by the behavior of opponent B, which in turn modifies the action possibilities of combatant A and so on and so forth [2, 3]. The two combatting athletes can be described as a single dynamical system (Fig. 1). This system self-organizes into (meta-)stable states as a result of the local-level behavior of both athletes aiming to successfully attack and defend under dynamic constraints, which emerge and decay during the fight [5]. In this approach, skilled behavior is the combatant’s ability to manipulate and take advantage of the (in)stability of the system as a whole.

To bolster these claims, we start with a brief explanation of the ecological dynamics approach, its application to social interaction, and the development of the concept of interpersonal synergies. We argue that adopting a synergetic approach to combat sports is necessary to truly capture the richness of the behaviors emerging when two athletes engage in combative interaction, a perspective that has largely remained out of scope with the typical individual-level analyses. Accordingly, the main aim of this work is to conceptualize combat as a social synergy using an ecological dynamics framework. To evaluate the extent to which our claims are supported by the literature, we review experimental work in combat sports. The final section delineates key issues for further research, for example, our understanding of skill and learning, and discusses implications of a social synergy perspective for future combat sports research and practice.

## The Ecological Dynamics Approach to Interpersonal Interactions

Contemporary understanding of how the behavior of one person influences that of another has been dominated by the cognitivist approach. This line of research focused mainly on the individual mind and how they infer what is going on in the minds of others using perceptual information generated in speech, movement postures, and/or facial expressions [15–18]. In this approach, understanding interpersonal interactions is underpinned by internal representations of the interactions. However, this type of research has been criticized for (1) focusing mainly on the cognitions of individuals within a social context, rather than on the interaction itself; (2) studying individuals passively observing others rather than actively interacting with them; and (3) emphasizing the use of discrete (verbal) knowledge of others over the use of dynamic, continuous information generated by others (i.e., invariants in optical and acoustic flows or inertia tensor) [19–22]. Therefore, a shift has been advocated to a more direct approach to social interaction [19, 22–24]. This approach emphasizes that social meaning is not a discrete (verbal) interpretation of events constructed within the mind of an observer; instead social meaning exists within the world and can be perceived directly and continuously by (actively) engaging with the social environment [24, 25]. These ideas fit better with an ecological approach to perception [26]. Rather than creating detached, internal representations about the (social) environment, ecological psychology stresses the evolutionary need to act with and adapt to the (social) environment in functional ways. At the heart of the ecological approach is the direct relation between the animal and its environment in terms of action capabilities or *affordances*:

“The affordances of the environment are what it offers the animal, what it provides or furnishes, either for good or ill. The verb to afford is found in the dictionary, but the noun affordance is not. I [James Gibson] have made it up. I mean by it something that refers to both the environment and the animal in a way that no existing term does. It implies the complementarity of the animal and the environment” [26, p. 127].

Ecological psychology research has produced an extensive body of work focusing on the perception of affordances provided by inanimate objects and events of the environment, such as the sit-ability of chairs [27] or the walk-through-ability of apertures [28]. Application of affordance theory on interpersonal interaction inspired the study of social affordances, that is, the opportunities for action offered or shaped by other humans [10, 20, 29]. Like objects, other humans may or may not afford simple and discrete actions such as grabbing, lifting, or striking. However, others also afford more complex and interactive behavior such as learning, fighting, making music, or falling in love with [20, 30]. As Gibson noted, interactions with other humans gives rise to “the richest and most elaborate affordances of the environment” [26, p. 135]. Recently, researchers have increasingly directed attention to how our sociocultural environment facilitates the emergence of new and original affordances [31–34].

## Interpersonal Interaction as a Synergy

Affordances are not static but constantly emerge, evolve, and decay during person–environment interactions [10]. These dynamics are especially apparent during interpersonal interaction, where the actions of one person invite some behaviors of the second person while discouraging other actions [4, 19]. Marsh and colleagues call for the need to study interpersonal interaction as an emerging feature from the temporary coupling between two persons [14, 35, 36]. Individuals constantly co-adapt their behavior to each other within a social exchange, so that a new, joint perception–action system emerges, which has been coined an *interpersonal synergy* [13, 14]. Within an interpersonal synergy, the perception and action of the two individuals are mutually constrained and coupled:

“Each individual’s perception is coupled to his or her partner’s action as it is to his or her own, and each individual’s action alters their partner’s perception just as it alters his own” [14, p. 20].

In motor control, the concept of a synergy was first introduced by Bernstein [37] as a solution to the degrees of freedom (DOF) problem. Instead of proposing a central executive controlling all the body’s individual DOF to solve a motor problem, Bernstein proposed that redundant DOF are reciprocally coupled so that they control each other. These ideas were further formalized by Haken et al. [38] in what became known as the HKB model. This model conceptualizes human behavior at different levels of analysis as a dynamical system, whose structurally complex but coupled components self-organize into stable movement patterns or *attractor* states. For example, Haken et al. [38] modelled bimanual index finger movements as coupled oscillators moving either in-phase or anti-phase. A key aspect of dynamical systems is nonlinearity; small changes within any of the system’s constraints can cause sudden transitions from one state to the other [39]. For instance, at certain critical speeds, the bimanual index finger trajectories showed a transition from anti-phase to in-phase pattern. Interestingly, the HKB model can be extended to describe coordinated movement of different body segments not only within a person (*intrapersonal*) but also between different individuals (*interpersonal*) [40–42]. The framework of interpersonal or social synergies has been applied to describe coordinated dyadic behavior within various contexts, such as dialog [43], music [44], dancing [45], and sports [46].

Two key characteristics of any synergy are *dimensional compression* and *reciprocal compensation* [13, 47]. Dimensional compression relates to the reduction of DOF within the synergy as a result of the constraints through which they are coupled. Variables capturing the lower-dimensional, ordered state of a synergy are known as *order parameters* [39]. For instance, the separate movement trajectories of two index fingers in the experiments by Haken et al. [38] were captured by a single-order parameter describing the two fingers’ relative phase. Reciprocal compensation refers to the co-adaptation of different components of a synergy to each other, enabling the system to respond to perturbations and maintain global-level order through local-level co-adaptation, reducing the need for top-down control. Within a synergy, the dynamics of local-level components give rise to order at the global level. This higher-level order then goes on to act as a constraint on the local-level components of the synergy. This process has been termed *timescale enslavement* [47], as the more slowly changing global order both arises from and then goes on to constrain the local, faster-timescale dynamics.

## Combat as a Dynamical, Self-Organizing System

Over the last 2 decades, empirical support for modelling sport situations as self-organizing systems has started to emerge, for example, in (sub-phases of) team sports [48–52] and racket sports [46, 53–55]. We propose to also understand two athletes in combat as an interpersonal synergy. When two combatants engage in combat, their perceptions and actions become coupled and mutually constrain one another. From this perspective, combat can be analyzed at both a local and a global level. At the local level, two individuals pursue mutually exclusive goals. Both individuals in combat aim to score (e.g., throw, strike, submit) without being scored against (e.g., being thrown, struck, submitted). From the co-adaptation between the two athletes, a global coordination arises within the fight. Once a global structure within a fight is established, this goes on to constrain or *enslave* [47] further behaviors of the individual combatants. An effective description of the interactions between combatants requires approaches for capturing both “discrete movement” at the local level (e.g., modelled as “point attractors”) and rhythmic action at the global level (e.g., modelled as “limit cycles”) [56]. Relating these two levels of analysis is an important scientific challenge in combat sports [57].

Affordances for attack and defense emerge, evolve, and decay within a fight as a result of behaviors of the individual athletes. Note that within a one-on-one competitive situation, combat affordances are complementary (or nested); an opponent affords being hit not only when they are within striking distance but also when they would not be afforded to block or strike back [26]. To successfully execute or defend to a scoring technique within combat sports (e.g., a strike, throw, or submission), the body of the combatant must be positioned relative to the opponent in certain specific ways. Depending on constraints, this can lead to the emergence of more or less predictable behavior at local “fixed points.” Variables describing the relative spatiotemporal positioning of the two combatants, such as their relative distance, height, center of mass, orientation, or velocity, thus seem to be key to understanding combat affordances that emerge at certain fixed points such as the “strikeability”, “throwability”, or “submitability” of an opponent.

As both athletes simultaneously attempt to score points while preventing the other from doing so, we expect (closely matched) combatants to self-organize into largely *stable* fights where the perceived action capabilities of both athletes are balanced out; neither athlete perceives an opportunity to advance their chances of success that is not immediately anticipated or reacted to by a balancing movement of the opponent (i.e., reciprocal compensation). In such situations, potential order parameters describing the overall balance between athletes would be expected to be relatively stable. To advance, athletes should first put effort in destabilizing the system so that they may then guide it towards a new, more advantageous state. Dynamic systems theory predicts such destabilizations and transitions should be visible as respectively enhanced fluctuations and sudden changes in order parameters [39].

Both within racket sports and within one versus one subphases of team sports, the abilities to break or lead the symmetry (i.e., destabilize the system) have been identified as key to offensive performance, whereas the behavior of defending players should be aimed at maintaining or restoring symmetry (i.e., stabilizing the system) [50, 53]. We hypothesize that in agreement with findings in other sports, breaking and restoring symmetry are key to performance within combat sports. A noticeable difference between combat sports and other competitive sport dyads is that in combat both athletes are constantly switching between attack and defense—a switch that can readily take place whilst in the middle of an attack [1, 4, 9]. Within ball sports, there are clear roles of attackers and defenders. In racket sports, the dynamics of hunter and prey (or actor and reactor, see [46]) may change within the discrete timeframe of a single shot. However, within combat sports, these dynamics may change at any instant. For example, instead of restoring symmetry by evading a kick from their opponent, a defending karate athlete may also initiate a counterattack and suddenly gain the initiative over the fight. Kimmel and Rogler [4] consider the ability to successfully operate around these critical or *meta-stable* regions to be an essential element of expertise in combat sports. Metastability arises when a system (e.g., a fight) lingers near a critical point, where it might suddenly switch between two or more competing modes of action (e.g., a successful strike, an evaded strike or a counterattack) [58–60]. Accordingly, we expect combat experts to be better aware of their own action boundaries and exploit these by purposely acting in relatively unstable regions on the limits of what is possible. Kimmel and Rogler [4] refer to this quality as *brinkmanship*.

## Review of Experimental Work

**Fig. 2:**
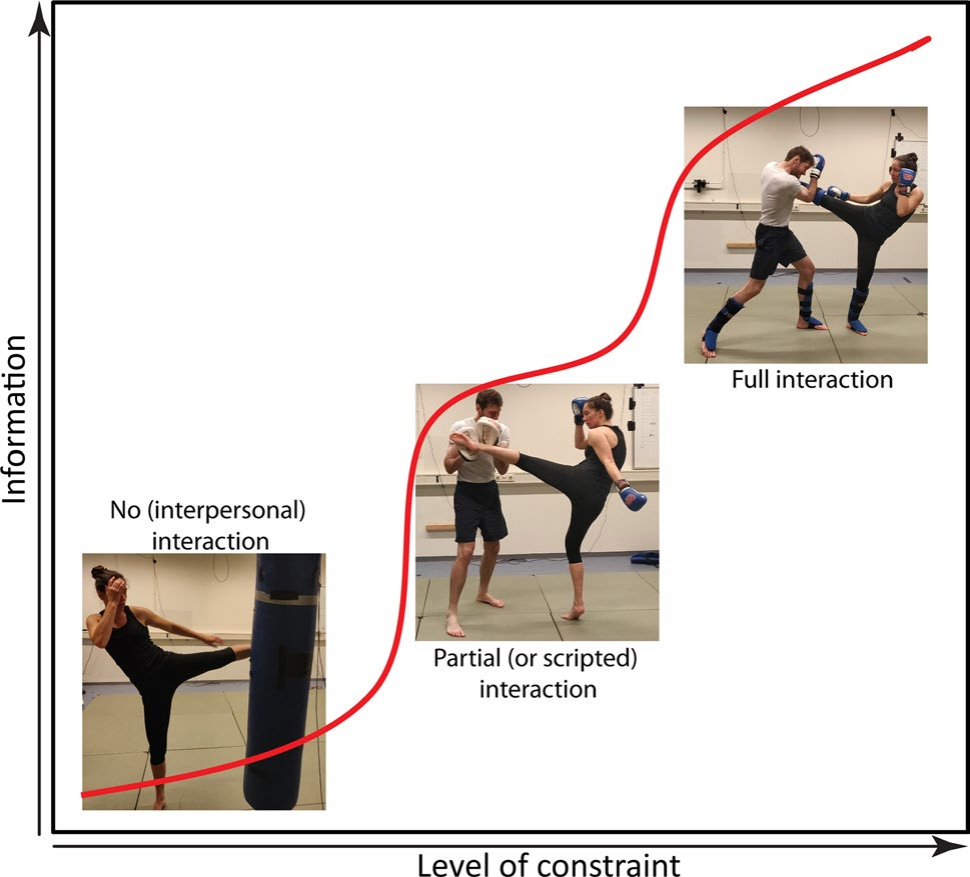
Informational complexity increases together with the level of interaction to which the combat task is constrained

In this section, we review empirical research on skilled behavior within combat sports and discuss these studies in light of the interpersonal synergy framework. Studies were categorized in three groups on the basis of the level of interaction allowed for within the experimental design. Accordingly, the first category of studies involved a single participant without a real opponent. The second group of studies involved an opponent whose behaviors were largely restricted and/or pre-described. A third group of studies allowed full interaction between two combatants as normally observed during free training (*sparring*) and in competition. Figure 2 exemplifies these study characteristics and implies a theoretical impact on the information available to individuals acting under these various constraints. Specifically, we expect that the least information is available under constraints with no interaction and the most information is available under constraints with full interaction [61, 62].

By reviewing these studies we aimed to (1) identify the extent to which the methods and findings in the current literature support the conceptualization of combat as a social synergy; (2) understand opportunities and limitations of the experimental paradigms to study the complex dynamics observed in one-on-one combat situations, and (3) identify key issues for further research.

### No Interaction

Expert–novice differences in visual search behavior have been studied under a video-based paradigm in French boxing [63] and karate [6]. Participants fitted with eye trackers watched video recordings of an opponent executing an offensive action and needed to anticipate the direction of the attack by moving a joystick [63] or by performing a defensive movement in front of the screen [6]. Both studies found experts focused longer and more centrally on the opponent’s body, whereas the gaze of novices was more dispersed and fixated more at the limbs. However, the video-based paradigm is limited in its representability because it breaks up the typical mutuality between perception and action [64]. For example, adequately responding to an incoming punch or kick (e.g., by blocking or counterattacking) is a motorically much more demanding task, with much more stringent time constraints, than simply moving a joystick [63] or moving in front of a screen “as if to avoid being struck” [6, p. 366].

In striking sports, acting at the right time from a proper distance is generally considered crucial for competitive success [65]. More experienced fencers have been found to better scale their perceived attack range to their action capabilities, that is, they better estimate the distance over which they can hit a (stationary) target than do their less experienced counterparts [66, 67]. In these experiments, participants were first asked to perceptually estimate the reachability of targets at different distances. These perceptual estimates were then compared with their actual maximum striking distance. Interestingly, elite fencers had lower actual striking distances then junior fencers, but they were more accurate in estimating their maximum striking distance [67]. This provides support for the suggestion that elite fencers are better attuned to their affordance boundaries than are junior fencers.

More work on striking affordances comes from Hristovski et al. [5], who adopted the ecological dynamics framework to study the emergent behavior of boxers punching a boxing bag from different distances. The results of this study showed that the perception and actualization of striking affordances (i.e., different types of punches) was scaled to action capabilities; different body-scaled distances (i.e., distances expressed in arm and leg span) afforded different types of strikes. In fact, Hristovski et al. [5] identified a meta-stable action distance that afforded multiple strikes. The authors hypothesized that actions produced from this meta-stable zone or region maximize the perceived efficiency and unpredictability of punching actions. At other distances (typically very close or far from the bag), punches are less efficient and more predictable. The near and far distances may thus be considered (relative) safe zones, where boxers run a low risk of being hit but are also unlikely to land a punch. At a medium distance, boxers may have more opportunities for attack, but they run higher risks of being hit themselves. Brinkmanship would thus be required to enter and successfully operate within this meta-stable area.

Initial work on single combat athletes thus started with studies on perceptual expertise disconnected from representative actions (i.e., video-based paradigms) but gradually evolved towards actively perceiving and controlling affordances. These studies support the notion of affordance-based control within combat sports regulating individual-level behavior. Within striking sports, body-scaled distance to the target has been identified as a key perceptual constraint on (perceived) action capabilities. Experts are suggested to be more sensitive to their action boundaries than less experienced combatants and hence better equipped to operate in meta-stable regions at the limits of their capabilities. However, as boxing bags or video-taped opponents do not (inter)act, these studies cannot establish whether and how co-adaptation of two combatants takes place, and whether two interacting combatants can be understood as a single interpersonal synergy.

### Partial or Scripted Interaction

A number of experimenters did include in-situ interaction between the two combatants but maintained experimental control by introducing scripted opponents (i.e., actors) and/or set roles. In-situ experiments on grip fighting in judo [68] and decision making in karate [7] have largely confirmed the expertise-related differences reported from video-based paradigms. Both studies included a standardized expert opponent who competed against all participants. An interesting aspect of the study by Milazzo et al. [7] was that the scripted attacker repeated the same attack every four actions but randomized the other attacks. Experts were more proficient in picking up and utilizing this repetitive pattern unfolding on a longer time scale than intermediate-skilled karate athletes (for similar findings in tennis, see Farrow and Reid [69]). They showed faster and more accurate responses on the repeated compared with the random attacks (after the sixth repetition), and verbal reports showed that the experts were more consciously aware of the repetitive attack pattern.

Some support for the utilization of information on even longer time scales was found by Sánchez-García et al. [70]. They studied adaptive behavior in practitioners of Krav Maga, a combat system that incorporates both striking and grappling techniques. Participants of different expertise levels were lined up to defend against one of the experimenters who acted as the attacker. In one group, the attacker was dressed as a boxer but attacked with a judo technique; in the other group, the attacker used a boxing punch while wearing a judo outfit. The results of the study, which were analyzed qualitatively, showed that all participants were initially surprised by the unexpected move. This suggests they had built strong expectations regarding the type of fighting related to outfit. However, experts and intermediates were better able than novices to functionally adapt to the situation after their initial surprise.

Caron et al. [1] adopted an interpersonal synergy perspective as their starting point in studying the effects of skill on the ability of individual aikido practitioners to co-adapt to each other’s actions and thereby maintain overall interpersonal coordination. Participants were paired together according to skill level and were randomly assigned a role of attacker or defender and asked to perform a prescribed offensive move and defensive reaction. The authors assessed three-dimensional kinematics of relevant effectors (wrist, elbow, and sternum) and assessed both interpersonal coordination, measured as movement synchronization between attacker and defender effector pairs, and intrapersonal coordination, measured as movement synchronization of the individual participant’s effectors. As an experimental manipulation, weights were attached to either the attacker’s or defender’s wrist. Results indicated that all participants co-adapted their intrapersonal (local) coordination to form stable interpersonal (global) behavioral patterns. However, skilled pairs demonstrated stronger coupling strengths and were better able to maintain their interpersonal coordination under the perturbations of the wrist-attached weights. They achieved this more stable interpersonal coordination through higher degrees of variation in intrapersonal movement organization. That is, expert dyads showed more adaptive flexibility to maintain global performance under changing constraints by reciprocally compensating to each other at the local level. This study neatly showed how dynamical systems methodology can be applied to analyses of interpersonal synchronization in combat situations. However, although the authors framed their experimental task as an example of a “competitive social motor activity”, the participants in this study were explicitly instructed to “perform the technique as a coordinated pair” [1, p. 257]. The studied task was thus actually a cooperative rather than a competitive task, raising doubt about the representativeness of the study for genuine competitive combat.

Research that adopted a scripted interaction approach thus led to further understanding of combat expertise. Results from these studies suggest combat experts can functionally co-adapt to their opponent to maintain interpersonal synchrony. However, the pre-assigned roles and/or movement patterns mean these studies do not account for the inherent nature of combat sports in which “two players must change continuously and instantaneously between offensive and defensive roles” [3, p. 2], requiring “the careful control of spatiotemporal parameters at the cost of potentially being hit by an attacker” [1, p. 256]. Therefore, an in-situ but controlled interaction approach still limits the possibilities of examining the exploitation of brinkmanship.

### Full Interaction

Only a few studies have favored a more representative task design above experimental control and have taken on the challenge of analyzing combat sports during interactions between two participants who were free to attack and defend. We recently adopted a full interaction approach to study the impact of full loss of vision in Paralympic judo [71]. Paralympic judo is controversial in that partially sighted and fully blind athletes all compete against each other within the same competitive class [72]. To put the current system to the test, we let able-sighted judo athletes compete in two simulation matches against the same opponent. In each match, one of the athletes fought blindfolded while the other fought fully sighted. Matches started with both athletes taking a grip on their opponent, according to para-judo rules. We found that athletes performed significantly worse (i.e., they scored less points) when fighting blindfolded. By comparing two matches between the same athletes, we were able to compare the impact of a constraint at the individual level on the stability of the system at the synergy level.

Maloney et al. [8] looked into the representativeness of taekwondo sparring in training compared with fighting in competition. They found that cognitive and affective demands (i.e., quantitative and qualitative assessments of mental effort, arousal, and anxiety) were lower during training than in (simulated) competition, and this was reflected in more predictable individual movement trajectories and larger interpersonal distances in training than in competition. Building on the frameworks of representative design [73] and affective learning design [74], the authors concluded that design of combat training should sample not only constraints shaping perceptual demands but also the cognitive and affective demands of competition. From a synergy perspective, we suggest that the participants in this study may have shown higher degrees of cooperation (i.e., lower competitiveness) and less willingness to operate in meta-stable regions within training, which resulted in stable and predictable behavioral patterns; within combat, increased variability in local-level behavior can be expected as individuals attempt to either break or restore symmetry, acting at the edges of their action boundaries under high perceptual, cognitive, and affective demands. Because athletes in training synergized more cooperatively, they formed more stable synergies at larger interpersonal distances than in competition, avoiding the meta-stable regions where brinkmanship can be developed.

To understand the basic dynamics of learning and synchronization in combat, Kijima et al. [2] recruited participants without prior combat sports experience to compete in a game of tag. In this game, both players have two tags attached to the sides of their hips and are instructed to catch and remove either of the opponent’s tags. The game is a simplification of the general aim of striking sports, which is to hit the opponent without being hit. The authors found that, over the course of ten trials against the same opponent, participants verbally reported improved tactical understanding of the game, which was reflected in higher degrees of movement synchronization and longer duration of the game. As the participants gained more experience in the game, their movements tended to self-organize into a stable anti-phase coupling; as one player stepped in (offensive action), the other stepped out (defensive reaction). These findings suggest synergies emerged, in which both components (i.e., combatants) reciprocally compensate for each other’s actions, leading to highly stable fights.

The emergence of synergetic behavior has also been studied within an actual combat sports context. The movement of pairs of expert *kendo* (Japanese sword fighting) players competing in simulated competitions tends to self-organize to maintain a critical interpersonal distance around either 2.7–2.8 or 1.0–1.1 m [3, 9]. The far distance was perceived to be an optimal distance balancing out the opportunity to step in for an attack, while still providing sufficient time to defend against an opponent’s attack. The close distance was described as a close-contact situation in which neither of the athletes can successfully land an attack (comparable with a clinch in boxing). These two distances (up close and far away) thus seemed to serve as the stable safe zones suggested by Hristovski et al. [5], whereas the distances in between reflect an unstable state from which athletes will either attack or reposition themselves. In their studies, Okumura et al. [3, 9] conceptualized interpersonal distance as a control parameter on the emerging behavioral patterns of the athletes (i.e., stepping velocities changing from in-phase to anti-phase around critical interpersonal distances). Local-level behavior (individual athletes stepping towards or away from each other) scales the control parameter up and down, thereby stabilizing or destabilizing the fight at the global level. In further work, Yamamoto et al. [57, 75] modelled the interactions of kendo combatants as a hybrid dynamical system to characterize both discrete (stepping maneuvers and striking opportunities) and cyclical behaviors (preferred interpersonal distances and velocities for attacking and defense). This hybrid system comprises both a higher, discrete module and a lower, continuous module connected through a feedback loop, which allows for “very complex, diverse, continuous human movement” [75, p. 6].

## Implications for Combat Research and Practice

Research into skilled behavior in combat sports appears to move gradually from individual-level analysis under experimentally controlled conditions toward the study of more representative behaviors that emerge from the dynamic interaction between two combatants. There is now some initial support for the idea that co-adaptation of two rivalling competitors leads to self-organization of the athlete dyad at a global level. In this section, we highlight some of the implications of this approach and identify a research agenda for further study.

### Modelling Combat as a Dynamical System

To demonstrate that the behavior of two actors involved in a combat sports task is synergetic, researchers would need to “use methods to quantify the collective state of the interpersonal or group dynamics that defined the phenomena in question” [47, p. 113]. The premise is that the full complexity of two athletes in combat can be captured by lower-dimensional order parameters:

“Instabilities open a path into theoretical modelling of the collective variable dynamics. In other words, they help us find the equations of motion. The idea is to map observed patterns onto attractors of the collective variable.” [39, p. 45]

**Fig. 3:**
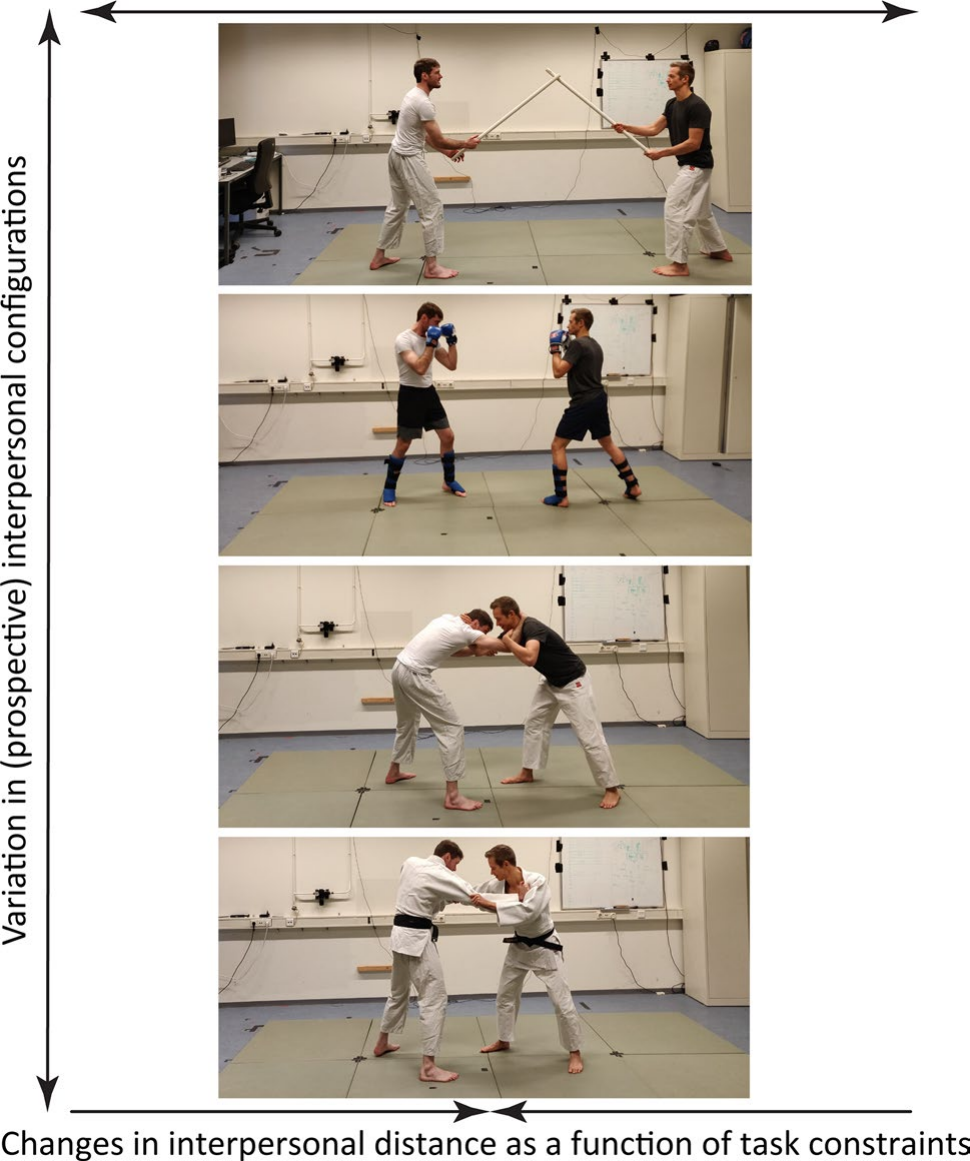
Under constraints imposed by different martial arts (i.e., use of weapons, allowed use of the limbs, or the use of different pieces of clothing), we can observe qualitatively distinct spatiotemporal configurations of each synergy. These changes in task constraints very likely lead to differences in the order parameters used to describe the (in)stability of each system. See text for discussion

Not many researchers systematically identified and assessed candidate order parameters. The relative phase of stepping behavior has been proposed as a potential order parameter in combat sports [1–3]. Yet, the extent to which different relative phase values relate to qualitatively distinct configurations of athletes observed across different combat sports has remained unclear (Fig. 3). For instance, in striking sports, managing the relative anterior-posterior distance seems to affect the (in)stability of these interpersonal systems [9]. In wrestling and judo, on the other hand, combatants try to “pick up” and throw their opponent to the ground – hence it can be advantageous to get under the center of mass of the opponent [76]. Therefore, we expect that more refined order parameters are needed to capture interpersonal coordination in combat, such as combatant’s relative orientation or relative center of mass.

Finding order parameters (or collective variables) goes hand in hand with identifying the control parameters of the system:

“You don’t really know you have a control parameter unless its variation causes qualitative change; qualitative change is necessary to identify collective variables unambiguously.” [39, p. 45]

Within striking sports (e.g., boxing, kendo), interpersonal distance appears to be a candidate control parameter that guides the system through different stable and unstable states [2, 3, 5]. Small changes in interpersonal distances have been found to cause sudden changes in the stability of fights. In these respects, interpersonal distance seems to be a variable generalizable across different sports, although the critical values around which phase transitions occur will likely be sport and context specific. For example, as depicted in Fig. 3, in kendo (where participants try to strike each other with a wooden sword), stable interpersonal distances are larger than in kickboxing (where participants can strike with the legs and arms). More broadly speaking, combat synergies appear to exhibit signs of s*elf-organized criticality* [77]. Without external tuning, the system organizes itself near critical points, where small variations in control parameters (e.g., interpersonal distance) can cause sudden bifurcations leading to success or failure for either athlete. Newell’s constraints model [78] might be applied to distinguish other personal (e.g., anthropometrics), environmental (e.g., size and shape of the combat area) and task constraints (e.g. rules of the game) on the self-organization of combat synergies. For example, Hristovski et al. [5] noted that, in boxing, besides interpersonal distance “other constraints like the defensive position of the arms of the opponent may regulate the attacker’s intentions in specific ways which requires further investigation” (p. 61). We expect that approaches examining the probabilities of different affordances at fixed points (e.g., such as examining state transition probabilities [57]) can reveal insights into the constraints on the emergence of affordances during combat. This should highlight important information sources for supporting emergent goals and, subsequently, how information sources may need to vary.

### Action Boundaries and Brinkmanship

Combat athletes need to constantly co-adapt their behavior to their opponent, outweighing the potential benefits and risks of their actions. To achieve this, they need to be highly sensitive to their own action boundaries and willing or daring to act in the meta-stable region close to these boundaries. To systematically assess the perception of action boundaries in combat sports, researchers should seek to (1) identify relevant information (on different time scales) that specify affordance boundaries, (2) compare the difference in information use in athletes from different skill levels, and (3) design training interventions to educate the attention of athletes to relevant information. Thus far, researchers examined the impact of skill between fights, comparing the behaviors of more and less skilled pairs of combatants [1, 9] but not the impact of skill within fights and/or how individual actions might manipulate the stability of the fight. An example of such a manipulation is the off-center effect found in soccer goalkeeping by Masters et al. [79]; by standing slightly off left or right of the goal center, goalkeepers can bias a penalty taker to shoot to the bigger side of the goal. Kimmel and Rogler [4] argued that similar tactics may be deployed by combat athletes by providing information for “false affordances”, evoking the opponent to certain actions that they could then take advantage of (i.e., deception).

### Learning Design

The central claim of this paper is that skilled behavior in combat sports emerges from the interaction between the two combatants. This position implies that skilled behavior should not be sought solely within the individual athlete but rather that the emergence of skilled action is distributed across the athlete–opponent interaction. From this perspective, questions might be asked about the effectiveness of many training methods traditionally employed within combat sports such as punching a bag or drilling techniques on a non-interacting training partner. Although we do not wish to claim that these practices do not further some aspects that are related to skill (e.g., these types of practice provide opportunities to explore the attractor space), we believe they do not entail skill in combat sport itself, such as is commonly conveyed. Alternatively, sparring against many different and quality opponents is generally considered to be a critical element within combat sports training. Athletes need to learn to quickly perceive and adapt to the constraints of a synergy they enter in a competition and even to changes on these constraints occurring within a single match (i.e., because of fatigue, or score progress). In professional boxing, the amount of competition is considerably lower than 50 years ago. Silver [80] argued that this has caused the skill level of professional boxers to decrease, even though they undertake much more sparring nowadays. Indeed, Maloney et al. [8] showed that sparring in training may often not be engaging enough to account for true synergistic action and the emergence of skilled behavior. This would imply that, to promote learning, athletes may need to increase the number of competitive fights they enter and/or increase the representativeness of sparring in training. We suggest that coaches should especially be concerned with finding ways to let athletes practice within meta-stable regions to promote the development of brinkmanship. For instance, coaches may limit the combat area in which athletes may move during sparring, so that athletes are constrained to practice at critical, meta-stable distances. The more popular phrase might be that individuals should be encouraged to operate “out of their comfort zone” [81] to ensure optimal learning and performance.

### Learning with Others

**Fig. 4:**
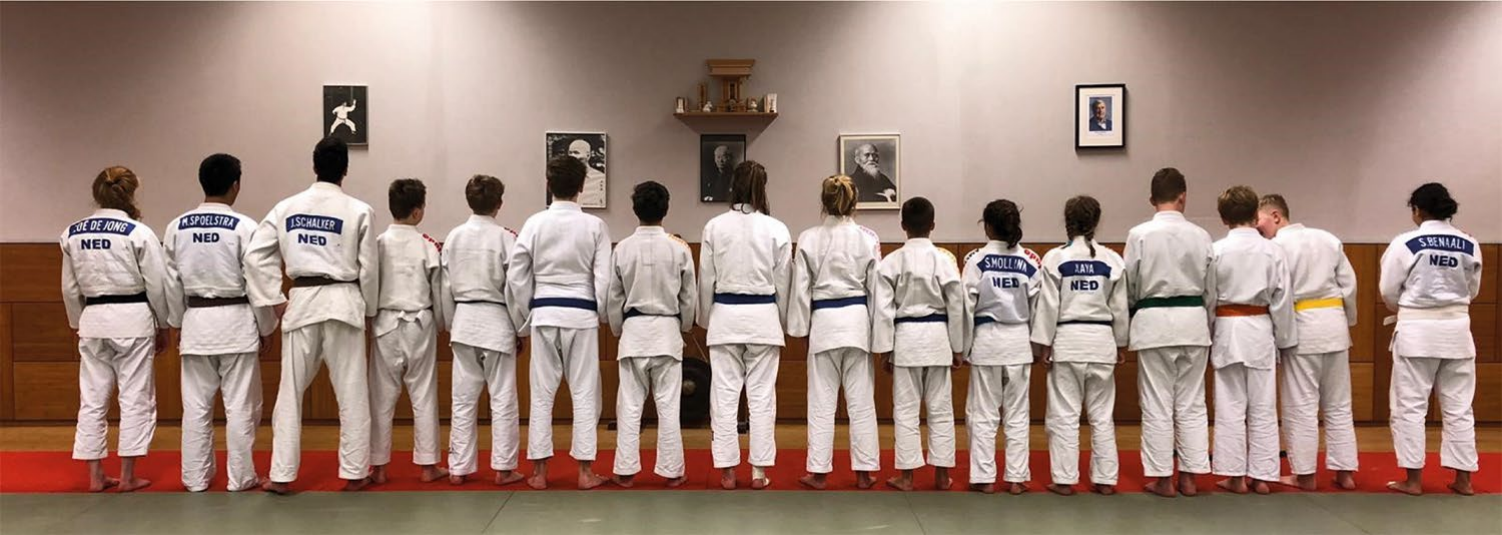
A group of Judo learners. As the belt colors change (from white/right to black/left), so each individual’s expertise level increases (and presumably so do action boundaries). Note also the variation in age, size, sex across individuals in the group

To learn, individuals do so together with others, who have a diversity of characteristics [82]. For example, individuals will vary from each other, perhaps in terms of expertise, age, anthropometrics, or sex (Fig. 4). During practice, learners must adapt to each other to achieve a (common) learning goal. This may mean that a more skilled athlete “comes down” to the level of the other or adjust their technique to the size of their partner. Another important consequence of learning with others is that it is expected to lead to a much larger range of actions being explored (i.e., the movement repertoire is increased) than if an individual were to practice alone [51]. Variability in practice is known to be beneficial for learning [12, 83–85]. Coaches should thus be aware that regular switching of training partners is likely to promote learning, even though athletes might tend to stick to their preferred training partner. Differences across group members may be advantageous because individuals must continuously learn to adapt to changing group demands, driving individuals to explore a broader range of approaches to achieve brinksmanship [86, 87]. Indeed, retrospective evidence indicates that elite athletes develop under conditions surrounded by other athletes, siblings, and coaches who constantly challenge them throughout their learning; they never really learn in isolation or under conditions of uniformity/stability [88–90]. In these respects, combat sports also represent an excellent research vehicle to address questions that are fundamental to understanding how learning is an essentially social practice and the mechanisms through which improved learning is supported.

## Conclusion

The ecological dynamics perspective offers a promising approach to further our understanding of skilled performance in combat sports and to assist coaches and athletes in promoting optimal training and learning. A review of the literature on skilled behavior in combat sports showed initial support for a conceptualization of combat dyads as a single dynamical system or interpersonal synergy. This approach implies that skilled behavior should not be sought solely within the individual athlete but rather that the emergence of skilled performance and learning is distributed across the athlete–opponent interaction. In particular, combat athletes require ‘brinkmanship’ to purposefully and accurately perceive and act near their action boundaries.

## References

[1] Caron RR, Coey CA, Dhaim AN, Schmidt RC (2017). Investigating the social behavioral dynamics and differentiation of skill in a martial arts technique. Hum Mov Sci..

[2] Kijima A, Kadota K, Yokoyama K, Okumura M, Suzuki H, Schmidt RC (2012). Switching dynamics in an interpersonal competition brings about “Deadlock” synchronization of players. PLoS One..

[3] Okumura M, Kijima A, Kadota K, Yokoyama K, Suzuki H, Yamamoto Y (2012). A critical interpersonal distance switches between two coordination modes in kendo matches. PLoS One.

[4] Kimmel M, Rogler CR (2018). Affordances in interaction: the case of Aikido. Ecol Psychol.

[5] Hristovski R, Davids K, Araújo D, Button C (2006). How boxers decide to punch a target: emergent behaviour in nonlinear dynamical movement systems. J Sports Sci Med.

[6] Williams AM, Elliott D (1999). Anxiety, expertise, and visual search strategy in Karate. J Sport Exerc Psychol..

[7] Milazzo N, Farrow D, Ruffault A, Fournier JF (2016). Do karate fighters use situational probability information to improve decision-making performance during on-mat tasks?. J Sports Sci.

[8] Maloney MA, Renshaw I, Headrick J, Martin DT, Farrow D (2018). Taekwondo fighting in training does not simulate the affective and cognitive demands of competition: implications for behavior and transfer. Front Psychol.

[9] Okumura M, Kijima A, Yamamoto Y (2017). Perception of affordances for striking regulates interpersonal distance maneuvers of intermediate and expert players in kendo matches. Ecol Psychol..

[10] Fajen BR, Riley MA, Turvey MT (2009). Information, affordances, and the control of action in sport. Int J Sport Psychol..

[11] Davids K, Glazier P, Araújo D, Bartlett R (2003). Movement systems as dynamical systems. Sports Med..

[12] Seifert L, Button C, Davids K (2013). Key properties of expert movement systems in sport. Sports Med..

[13] Riley MA, Richardson M, Shockley K, Ramenzoni VC (2011). Interpersonal synergies. Front Psychol..

[14] Marsh KL, Richardson MJ, Baron RM, Schmidt R (2006). Contrasting approaches to perceiving and acting with others. Ecol Psychol..

[15] Frith CD, Frith U (1999). Interacting minds—a biological basis. Science (New York, NY)..

[16] Frith CD (2008). Social cognition. Philos Trans R Soc Lond Ser B Biol Sci..

[17] Shantz CU (1975). The development of social cognition Review of child development research.

[18] Petri K, Bandow N, Salb S, Witte K (2019). The influence of facial expressions on attack recognition and response behaviour in karate kumite. Eur J Sport Sci..

[19] De Jaegher H, Di Paolo E, Gallagher S (2010). Can social interaction constitute social cognition?. Trends Cogn Sci..

[20] Valenti SS, Gold JM (1991). Social affordances and interaction I: Introduction. Ecol Psychol..

[21] McArthur LZ, Baron RM (1983). Toward an ecological theory of social perception. Psychol Rev..

[22] McGann M, De Jaegher H (2009). Self–other contingencies: enacting social perception. Phenomenol Cogn Sci..

[23] Sebanz N, Bekkering H, Knoblich G (2006). Joint action: bodies and minds moving together. Trends Cogn Sci..

[24] Schilbach L, Timmermans B, Reddy V, Costall A, Bente G, Schlicht T (2013). Toward a second-person neuroscience. Behav Brain Sci..

[25] Kono T (2009). Social affordances and the possibility of ecological linguistics. Integr Psychol Behav Sci..

[26] Gibson JJ (1979). The ecological approach to visual perception.

[27] Mark LS, Vogele D (1987). A biodynamic basis for perceived categories of action. J Mot Behav..

[28] Warren WH, Whang S (1987). Visual guidance of walking through apertures: body-scaled information for affordances. J Exp Psychol Hum Percept Perform..

[29] Hettinga FJ, Konings MJ, Pepping G-J (2017). The science of racing against opponents: affordance competition and the regulation of exercise intensity in head-to-head competition. Front Physiol..

[30] Rietveld E, de Haan S, Denys D (2013). Social affordances in context: what is it that we are bodily responsive to?. Behav Brain Sci.

[31] Glăveanu VP (2014). Distributed creativity: thinking outside the box of the creative individual.

[32] Immonen T, Brymer E, Orth D, Davids K, Feletti F, Liukkonen J (2017). Understanding action and adventure sports participation—an ecological dynamics perspective. Sports Med Open..

[33] Rietveld E, Kiverstein J (2014). A rich landscape of affordances. Ecol Psychol..

[34] Withagen R, van der Kamp J (2018). An ecological approach to creativity in making. New Ideas Psychol..

[35] Marsh KL, Richardson MJ, Schmidt RC (2009). Social connection through joint action and interpersonal coordination. Top Cogn Sci..

[36] Schmidt RC, Richardson MJ (2008). Dynamics of interpersonal coordination. Coordination: neural, behavioral and social dynamics.

[37] Bernstein NA (1967). The co-ordination and regulation of movement.

[38] Haken H, Kelso JS, Bunz H (1985). A theoretical model of phase transitions in human hand movements. Biol Cybern..

[39] Kelso JAS (1995). Dynamic patterns: the self-organization of human brain and behavior.

[40] Black DP, Riley MA, McCord CK (2007). Synergies in intra-and interpersonal interlimb rhythmic coordination. Motor Control..

[41] Ramenzoni VC, Davis TJ, Riley MA, Shockley K, Baker AA (2011). Joint action in a cooperative precision task: nested processes of intrapersonal and interpersonal coordination. Exp Brain Res..

[42] Richardson MJ, Marsh KL, Baron RM (2007). Judging and actualizing intrapersonal and interpersonal affordances. J Exp Psychol Hum Percept Perform..

[43] Fusaroli R, Rączaszek-Leonardi J, Tylén K (2014). Dialog as interpersonal synergy. New Ideas Psychol..

[44] Ragert M, Schroeder T, Keller P (2013). Knowing too little or too much: the effects of familiarity with a co-performer’s part on interpersonal coordination in musical ensembles. Front Psychol.

[45] Torrents C, Hristovski R, Coterón J, Ric Á, Passos P, Davids K, Chow JY (2016). Interpersonal coordination in contact improvisation dance. Interpersonal coordination and performance in social systems.

[46] McGarry T, Anderson DI, Wallace SA, Hughes MD, Franks IM (2002). Sport competition as a dynamical self-organizing system. J Sports Sci..

[47] Eiler BA, Kallen RW, Richardson MJ, Vallacher RV, Read SJ, Nowak A (2017). Interaction-dominant dynamics, timescale enslavement, and the emergence of social behavior. Computational social psychology.

[48] Bourbousson J, Sève C, McGarry T (2010). Space–time coordination dynamics in basketball: Part 1. Intra- and inter-couplings among player dyads. J Sports Sci.

[49] Passos P, Araújo D, Davids K, Gouveia L, Milho J, Serpa S (2008). Information-governing dynamics of attacker–defender interactions in youth rugby union. J Sports Sci..

[50] Duarte R, Araújo D, Davids K, Travassos B, Gazimba V, Sampaio J (2012). Interpersonal coordination tendencies shape 1-vs-1 sub-phase performance outcomes in youth soccer. J Sports Sci..

[51] Silva P, Garganta J, Araújo D, Davids K, Aguiar P (2013). Shared knowledge or shared affordances? Insights from an ecological dynamics approach to team coordination in sports. Sports Med..

[52] Passos P, Araújo D, Davids K (2013). Self-organization processes in field-invasion team sports. Sports Med..

[53] McGarry T, De Poel HJ, Passos P, Davids K, Chow JY (2016). Interpersonal coordination in competitive sports contests: racket sports. Interpersonal coordination and performance in social systems.

[54] Palut Y, Zanone P-G (2005). A dynamical analysis of tennis: concepts and data. J Sports Sci..

[55] Pereira TJC, van Emmerik REA, Misuta MS, Barros RML, Moura FA (2018). Interpersonal coordination analysis of tennis players from different levels during official matches. J Biomech..

[56] Nalepka P, Lamb M, Kallen RW, Shockley K, Chemero A, Saltzman E (2019). Human social motor solutions for human–machine interaction in dynamical task contexts. Proc Natl Acad Sci.

[57] Yamamoto Y, Kijima A, Okumura M, Yokoyama K, Gohara K (2019). A switching hybrid dynamical system: toward understanding complex interpersonal behavior. Appl Sci..

[58] Kelso JAS (2012). Multistability and metastability: understanding dynamic coordination in the brain. Philos Trans R Soc Lond Ser B Biol Sci..

[59] Pinder RA, Davids K, Renshaw I (2012). Metastability and emergent performance of dynamic interceptive actions. J Sci Med Sport..

[60] Hristovski R, Davids KW, Araujo D, Araujo D, Ripoll H, Raab M (2009). Information for regulating action in sport : metastability and emergence of tactical solutions under ecological constraints. Perspectives on cognition and action in sport.

[61] Davids K, Araújo D, Hristovski R, Passos P, Chow JY. Ecological dynamics and motor learning design in sport. Skill Acquisit Sport Res Theory Pract. 2012;112–30.

[62] Davids K, Araújo D, Seifert L, Orth D, Baker J, Farrow D (2015). Expert performance in sport: an ecological dynamics perspective. Routledge handbook of sport expertise.

[63] Ripoll H, Kerlirzin Y, Stein J-F, Reine B (1995). Analysis of information processing, decision making, and visual strategies in complex problem solving sport situations. Hum Mov Sci..

[64] Van der Kamp J, Dicks M, Navia JA, Noël B (2018). Goalkeeping in the soccer penalty kick. German J Exerc Sport Res.

[65] Roi GS, Bianchedi D (2008). The science of fencing. Sports Med..

[66] Chen YH, Liu YT (2015). To touch or not to touch? Fencers’ estimate of their reachability. Int J Sport Psychol..

[67] Turner AN, Marshall G, Noto A, Chavda S, Atlay N, Kirby D (2017). Staying out of range: increasing attacking distance in fencing. Int J Sports Physiol Perform..

[68] Piras A, Pierantozzi E, Squatrito S (2014). Visual search strategy in Judo fighters during the execution of the first grip. Int J Sports Sci Coach..

[69] Farrow D, Reid M (2012). The contribution of situational probability information to anticipatory skill. J Sci Med Sport..

[70] Sánchez-García R, Villaroya-Gil Á, Elrio-Lopez A (2016). Manipulating task constraints of situated normativity to study decision making in Krav Maga. Int J Sport Psychol..

[71] Krabben KJ, van der Kamp J, Mann DL (2018). Fight without sight: the contribution of vision to judo performance. Psychol Sport Exerc..

[72] Krabben KJ, Ravensbergen RHJC, Nakamoto H, Mann DL (2019). The development of evidence-based classification of vision impairment in Judo: a Delphi study. Front Psychol.

[73] Pinder RA, Davids K, Renshaw I, Araújo D (2011). Representative learning design and functionality of research and practice in sport. J Sport Exerc Psychol..

[74] Headrick J, Renshaw I, Davids K, Pinder RA, Araújo D (2015). The dynamics of expertise acquisition in sport: the role of affective learning design. Psychol Sport Exerc..

[75] Yamamoto Y, Yokoyama K, Okumura M, Kijima A, Kadota K, Gohara K (2013). Joint action syntax in Japanese martial arts. PLoS One..

[76] Imamura RT, Hreljac A, Escamilla RF, Edwards WB (2006). A three-dimensional analysis of the center of mass for three different judo throwing techniques. J Sports Sci Med.

[77] Bak P, Tang C, Wiesenfeld K (1987). Self-organized criticality: An explanation of the 1/f noise. Phys Rev Lett..

[78] Newell KM, Wade MG, Whiting HTA (1986). Constraints on the development of coordination. Motor development in children: aspects of coordination and control.

[79] Masters RSW, van der Kamp J, Jackson RC (2007). Imperceptibly off-center goalkeepers influence penalty-kick direction in soccer. Psychol Sci..

[80] Silver M (2008). The arc of boxing: the rise and decline of the sweet science.

[81] Brown M (2008). Comfort zone: model or metaphor?. Austr J Outdoor Educ..

[82] Orth D, van der Kamp J, Button C (2019). Learning to be adaptive as a distributed process across the coach-athlete system: situating the coach in the constraints-led approach. Phys Educ Sport Pedagogy..

[83] Orth D, van der Kamp J, Memmert D, Savelsbergh G (2017). Creative motor actions as emerging from movement variability. Front Psychol.

[84] Schöllhorn WI, Mayer-Kress G, Newell KM, Michelbrink M (2009). Time scales of adaptive behavior and motor learning in the presence of stochastic perturbations. Hum Mov Sci..

[85] Magill RA, Hall KG (1990). A review of the contextual interference effect in motor skill acquisition. Hum Mov Sci..

[86] Baldauf SA, Engqvist L, Weissing FJ (2014). Diversifying evolution of competitiveness. Nat Commun..

[87] Wright TF, Eberhard JR, Hobson EA, Avery ML, Russello MA (2010). Behavioral flexibility and species invasions: the adaptive flexibility hypothesis. Ethol Ecol Evol..

[88] Jones RL, Edwards C, Viotto Filho IT (2016). Activity theory, complexity and sports coaching: an epistemology for a discipline. Sport Educ Soc..

[89] Phillips E, Davids K, Renshaw I, Portus M (2010). Expert performance in sport and the dynamics of talent development. Sports Med..

[90] Wormhoudt R, Savelsbergh GJ, Teunissen JW, Davids K (2017). The athletic skills model: optimizing talent development through movement education.

